# Marine Derived Natural Products: Emerging Therapeutics Against Herpes Simplex Virus Infection

**DOI:** 10.3390/biom16010100

**Published:** 2026-01-07

**Authors:** Vaibhav Tiwari, James Elste, Chunyu Wang, Fuming Zhang

**Affiliations:** 1 Department of Microbiology & Immunology, College of Graduate Studies, Midwestern University, Downers Grove, IL 60515, USA; 2 Departments of Biological Science, Chemistry and Chemical Biology, Center for Biotechnology and Interdisciplinary Studies, Rensselaer Polytechnic Institute, Troy, NY 12180, USA; 3 Department of Chemical and Biological Engineering, Center for Biotechnology and Interdisciplinary Studies, Rensselaer Polytechnic Institute, Troy, NY 12180, USA

**Keywords:** herpes simplex virus, HSV entry, virus host cell interactions, marine derived sulfated glycans, viral entry inhibitors, antiviral, anti-inflammatory

## Abstract

Herpes simplex viruses (HSV-1 and HSV-2) are highly prevalent human pathogens that establish lifelong latency in sensory neurons, posing a persistent challenge to global public health. Their clinical manifestations range from mild, self-limiting orolabial lesions to severe, life-threatening conditions such as disseminated neonatal infections, focal encephalitis, and herpetic stromal keratitis, which can lead to irreversible corneal blindness. Beyond direct pathology, HSV-mediated genital ulcerative disease (GUD) significantly enhances mucosal susceptibility to HIV-1 and other sexually transmitted infections, amplifying co-infection risk and disease burden. Despite decades of clinical reliance on nucleoside analogues such as acyclovir, the therapeutic landscape has stagnated with rising antiviral resistance, toxicity associated with prolonged use, and the complete inability of current drugs to eliminate latency or prevent reactivation continue to undermine effective disease control. These persistent gaps underscore an urgent need for next-generation antivirals that operate through fundamentally new mechanisms. Marine ecosystems, the planet’s most chemically diverse environments, are providing an expanding repertoire of antiviral compounds with significant therapeutic promise. Recent discoveries reveal that marine-derived polysaccharides, sulfated glycans, peptides, alkaloids, and microbial metabolites exhibit remarkably potent and multi-targeted anti-HSV activities, disrupting viral attachment, fusion, replication, and egress, while also reshaping host antiviral immunity. Together, these agents showcase mechanisms and scaffolds entirely distinct from existing therapeutics. This review integrates emerging evidence on structural diversity, mechanistic breadth, and translational promise of marine natural products with anti-HSV activity. Collectively, these advances position marine-derived compounds as powerful, untapped scaffolds capable of reshaping the future of HSV therapeutics.

## 1. Introduction

**Figure 1:**
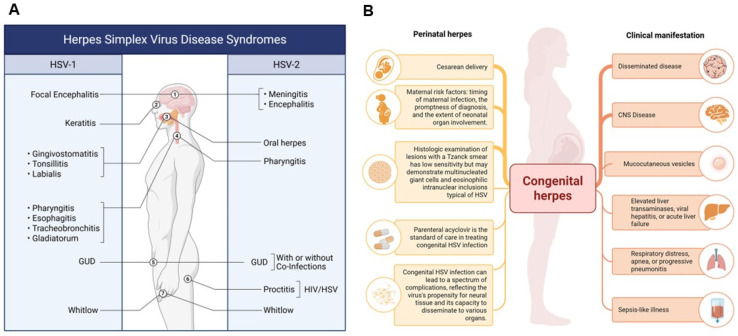
Epidemiology and Clinical Manifestations of HSV-1 and HSV-2. (**A**) The figure depicts the spectrum of infections caused by HSV-1 and HSV-2, including genital ulcerative disease (GUD) attributable to HSV-1 in adults. While HSV-1 has historically been associated with oral lesions, it is increasingly implicated in genital infections due to changing epidemiological trends. (**B**) Perinatal and Clinical Implications of HSV-2 Infection. This figure highlights perinatal HSV-2 infections, which primarily affect neonates. Clinical outcomes in infants depend on factors such as the timing of maternal infection and mode of delivery. Although HSV-2 is traditionally associated with genital lesions, it can occasionally cause oral infections. HSV-2 infections are often accompanied by co-infections with *Treponema pallidum* (syphilis), *Neisseria gonorrhoeae* (gonorrhea), and *Chlamydia trachomatis*, contributing to the broader spectrum of genital ulcerative diseases (GUDs). Additionally, HSV-2 increases susceptibility to and co-transmission with HIV. Collectively, these panels illustrate the evolving and overlapping clinical presentations of HSV-1 and HSV-2 across different populations and anatomical sites. Created in BioRender. Tiwari, V. https://BioRender.com/nv581og (accessed on 31 December 2025).

**Figure 2:**
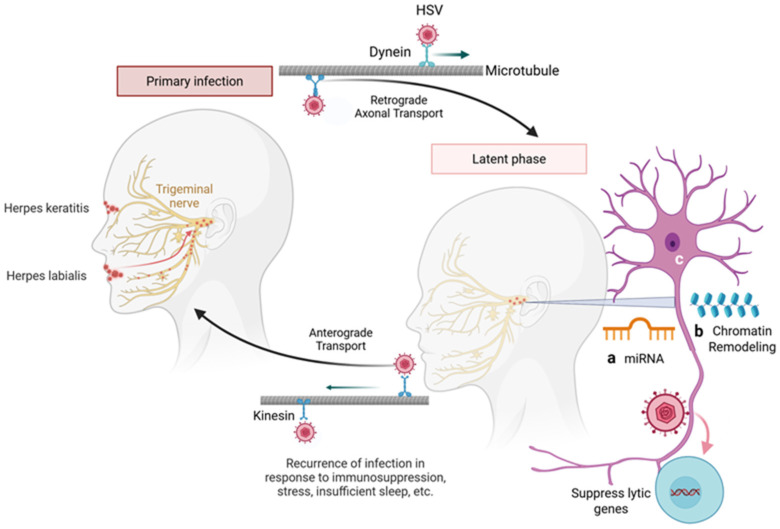
Herpes simplex virus (HSV) employs latency as a key mechanism to evade the immune system. Following primary infection at mucocutaneous surfaces, HSV travels retrogradely along sensory neurons to the peripheral ganglia using microtubule bound dynein motors, where virus establishes lifelong latency. Reactivation of the virus can occur in response to physiological, hormonal, or immunosuppressive stress, leading to anterograde transport using kinesin motors on the microtubule filaments back to the site of primary infection and the development of painful vesicular lesions. During latency, particularly in neuronal tissues, several factors contribute to the maintenance of viral dormancy. These include MicroRNAs (miRNAs) in which both host and viral miRNAs regulate gene expression to suppress lytic cycle genes and maintain latency (a). Similarly, chromatin remodeling such as epigenetic modifications (histone methylation and acetylation), create a repressive chromatin state over viral lytic gene (b). Finally, there are limited neuronal transcription factors necessary for HSV lytic replication, favoring a latent state. Together, these mechanisms underscore the intricate host-virus interplay that supports HSV persistence and immune evasion. Created in BioRender. Tiwari, V. https://BioRender.com/ggm2s52 (accessed on 31 December 2025).

Herpes simplex viruses (HSV-1 and HSV-2) are enveloped, double-stranded DNA viruses belonging to the *Herpesviridae* family and classified within the *Alphaherpesvirinae* subfamily [1]. HSV-1 infections are primarily associated with orofacial and cutaneous lesions above the waist, while HSV-2 predominantly affects the genital and mucocutaneous regions of sexually active individuals [2]. However, both viral types can infect either anatomical site and can cause severe neonatal herpes infections (Figure 1A,B) [3]. Following primary infection, HSV establishes lifelong latency within sensory neurons, characterized by epigenetic silencing of lytic genes and selective transcription of latency-associated transcripts (LATs) [4,5,6]. The principal site of latency for HSV-1 is the trigeminal ganglion, whereas HSV-2 typically establishes latency in the sacral root ganglia. Periodic reactivation, often triggered by physiological stress, immunosuppression, or environmental stimuli, results in recurrent lesions or asymptomatic viral shedding (Figure 2) [7].

HSV are highly prevalent human infections, with worldwide seroprevalence reaching approximately 90%. HSV-1 is the most common, with early childhood acquisition in developing countries and delayed exposure until adolescence or adulthood in developed regions; prevalence shows minimal increase with age and is similar between men and women [8,9,10]. In contrast, HSV-2 is primarily sexually transmitted, with prevalence varying by region, sexual activity, and demographics, reaching >50% in some African populations [11,12]. Women are at higher risk of HSV-2 infection than men, and African Americans demonstrate higher prevalence compared with whites and Asians [13,14]. HSV-2 infection also significantly increases susceptibility to HIV, likely due to mucosal ulceration and recruitment of CD4+ target cells, underscoring its public health importance [15,16,17]. With shifting epidemiology, HSV-1 increasingly causes orolabial infections, while HSV-2 traditionally genital can also present as oral ulcers, reflecting changes in transmission patterns and clinical presentation [18].

Congenital HSV infections are associated with significant neurological and systemic complications due to the virus’s neurotropic nature and its capacity to disseminate across multiple organ systems (Figure 1B) [19]. Central nervous system involvement, particularly in the form of herpetic encephalitis, can result in long-term neurological deficits, developmental delay, or cognitive impairment [20,21]. Ocular complications such as herpetic stromal keratitis and chorioretinitis may cause irreversible vision loss, while cutaneous manifestations can progress to disseminated infection [22,23]. Furthermore, systemic involvement of the liver, lungs, and adrenal glands can contribute to multiorgan dysfunction [24]. Neonatal HSV infection may also precipitate preterm labor, with high risks of morbidity and mortality despite current antiviral interventions [25].

Although significant progress has been made in understanding HSV biology, no licensed vaccine has yet achieved sterilizing immunity [26]. Current therapeutic regimens rely predominantly on nucleoside analogues such as acyclovir (ACV) and valacyclovir, which act as competitive inhibitors of viral DNA polymerase, suppressing viral genome replication and propagation [27]. However, the increasing prevalence of ACV-resistant strains, arising from mutations in the UL23 (thymidine kinase; TK) or UL30 (DNA polymerase) genes, coupled with limited bioavailability and potential cytotoxicity, underscores the urgent need for novel antiviral agents with distinct molecular targets and improved pharmacokinetic and pharmacodynamic profiles [28,29,30,31,32]. Moreover, prolonged use of antiviral and anti-inflammatory drugs can lead to severe side effects and further drive the emergence of drug-resistant strains [33,34]. Consequently, the development of new anti-herpes agents with high efficacy against resistant viral strains remains a critical priority.

**Figure 3:**
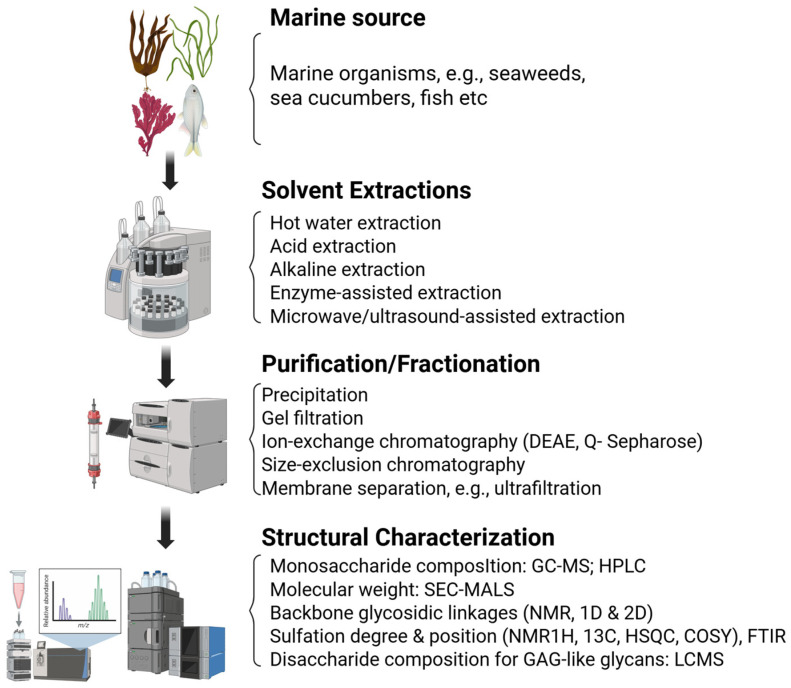
Schematic overview of the workflow for the structural and functional characterization of marine-derived sulfated glycans. The process begins with the extraction and purification of glycans from marine sources, followed by compositional analysis using techniques such as monosaccharide profiling and sulfate content quantification. Advanced structural elucidation is performed using methods like NMR spectroscopy and mass spectrometry. Finally, bioactivity assays including antiviral and anti-inflammatory screening platforms are conducted to evaluate the functional properties of the characterized glycans. Created in BioRender. Tiwari, V. https://BioRender.com/6p4524k (accessed on 31 December 2025).

The marine environment, which encompasses nearly half of global biodiversity, represents a prolific source of structurally diverse and biologically active metabolites [35,36,37,38]. Over 35,000 marine-derived natural products have been identified from microorganisms, algae, sponges, corals, and other invertebrates, approximately half of which exhibit measurable biological activity [39,40]. These compounds include sulfated polysaccharides, sulfated glycans, alkaloids, peptides, terpenoids, and polyketides, often featuring unique structural motifs such as halogenation, sulfation, and atypical glycosidic linkages, which confer distinctive pharmacological properties [41,42,43]. Marine-derived glycans are isolated and purified using advanced analytical approaches, including monosaccharide profiling and sulfate quantification. Their structures are resolved through Nuclear Magnetic Resonance (NMR) spectroscopy and mass spectrometry, and their functional properties are evaluated via antiviral and anti-inflammatory assays, providing insights into structure–activity relationships (Figure 3). Marine sulfated polysaccharides, such as carrageenans, fucoidans, and ulvans, have demonstrated a potent anti-herpes simplex virus (HSV) activity by inhibiting viral adsorption and viral entry via competitive binding to the host cell surface heparan sulfate (HS)-interacting viral glycoproteins gB and gC [42]. Additionally, several marine peptides and alkaloids act post-entry by interfering with intracellular replication complexes or modulating host antiviral pathways, including Protein Kinase R (PKR), Nuclear factor kappa-light-chain-enhancer of activated B cells (NF-κB), and interferon-stimulated gene (ISG) signaling [44,45].

In our recent study, we identified four marine-derived sulfated glycans—RPI-27 (fucoidan), FCS-Pg (fucosylated chondroitin sulfate from sea cucumber, *Pearsonothuria graeffe*), FCS-Ib (fucosylated chondroitin sulfate from sea cucumber, *Isostichopus badionotus*) and Rhamnan sulfate (RS) which exhibited potent, dose-dependent inhibition of HSV-1 entry at non-toxic concentrations [46]. Notably, the inhibitory activity of RS and FCS-Ib was significantly enhanced when pre-incubated with the HSV virions compared to pre-incubation with target cells. Further evaluation revealed that RS and FCS-Ib also effectively blocked virus-cell fusion and cell-to-cell spread, as evidenced by reduced HSV-1 glycoproteins (gB, gD, gH, gL)-mediated fusion and decreased plaque formation [46]. Surface Plasmon Resonance (SPR) analysis later confirmed preferential binding of these glycans to HSV-1 gD, supporting a mechanism of action at the viral entry step [46]. Interestingly, antiviral effects of sulfated glycans were also observed in post-infection models, suggesting their multivalent interactome may engage cellular host proteins, particularly HS-binding growth factors, thereby impacting viral replication [46,47]. These findings underscore the potential of marine-derived compounds as novel therapeutic agents against viral infections [48,49,50].

Given their structural diversity and mechanistic versatility, isolation and characterization of marine sulfated glycans targeting multiple steps of the viral life cycle remain highly desirable [51]. Accordingly, marine organisms represent a promising reservoir for antiviral drug development [52]. This review provides a comprehensive analysis of recent advances in the discovery and mechanistic characterization of marine-derived anti-HSV compounds, with particular emphasis on sulfated polysaccharides and secondary metabolites. Key focus areas include structure activity relationships, molecular targets, and mechanisms of viral inhibition, as well as the translational potential of these bioactive compounds as next-generation antivirals or adjuvants in combination therapies. Perspectives on future directions in marine-based antiviral drug discovery are also discussed.

## 2. The Medical Need—Challenges with Existing Anti-HSV Drugs

HSV infections affect a substantial portion of the global population, contributing to recurrent outbreaks and increasing susceptibility to HIV [16]. These conditions impose significant challenges for individuals with multiple other sexually transmitted infections (STIs) and strain healthcare systems worldwide. Additionally, the aging population, living longer than ever before, has seen a rise in the prevalence of dementia among older adults, further compounding the public health burden [53]. Current antiviral therapies, primarily nucleoside analogs like acyclovir (ACV) and its derivatives, have critical limitations, including poor central nervous system (CNS) penetration, restricted efficacy against latent infections, associated side effects with the long term medications, and the emergence of ACV-resistant strains [28,29,30,32,33,34]. Current management of HSV infections relies primarily on three classes of antiviral agents: acyclic nucleoside analogs (e.g., acyclovir, valacyclovir), acyclic nucleotide analogs (e.g., cidofovir, adefovir dipivoxil), and pyrophosphate inhibitors (e.g., foscarnet) [54]. These agents inhibit HSV DNA replication. ACV, approved in 1977, is phosphorylated by viral thymidine kinase to acyclovir monophosphate (ACV-MP), which is subsequently converted by cellular kinases to the active triphosphate form (ACV-TP) that competitively inhibits viral DNA polymerase [55]. Despite its efficacy, ACV has limited oral bioavailability, a short plasma half-life [56], and restricted central nervous system (CNS) penetration due to hydrophilicity and active efflux from the brain. In patients with herpesvirus CNS infections, blood–brain barrier disruption can increase ACV concentrations, which may lead to acyclovir-induced neuropsychiatric symptoms (AINS) associated with high cerebrospinal fluid levels of the metabolite 9-carboxymethoxymethylguanine (CMMG) [57]. Valacyclovir, an oral prodrug of ACV, shares similar efficacy and safety profiles, although rare psychiatric adverse effects, including psychosis, have been reported. Cidofovir (CDV), an acyclic nucleotide analog, does not require viral kinase for activation and remains active against HSV and varicella-zoster virus (VZV) strains lacking thymidine kinase [58]. Foscarnet, a pyrophosphate analog, inhibits viral DNA polymerase by targeting its pyrophosphate-binding site and is effective against thymidine kinase-deficient strains [59]. Prolonged or high-dose antiviral therapy can cause adverse effects. ACV commonly leads to malaise, while less frequent complications include infusion-site inflammation, phlebitis, nausea, vomiting, transaminitis, and cutaneous reactions such as Stevens–Johnson syndrome. Pediatric patients may experience decreased hemoglobin and neutrophil counts [60]. Acute kidney injury (AKI), due to crystal-induced nephropathy, is a serious concern during intravenous therapy [61]. Antiviral-resistant HSV is another emerging clinical problem, particularly in immunocompromised populations. Although ACV-resistant HSV is rare (<1%) in immunocompetent patients, resistance is more likely in immune-privileged sites such as the cornea or in patients undergoing hematopoietic stem cell transplantation (HSCT), where prolonged therapy increases the risk of resistant strains [28,29,30,32,33,34].

A novel class of chemical inhibitors, helicase-primase inhibitors (HPIs), specifically targets the heterotrimeric helicase-primase complex, a critical enzyme required for HSV DNA replication [62]. By disrupting this complex, HPIs halt viral replication prior to substantial DNA synthesis, providing antiviral efficacy even during later stages of infection. Unlike nucleoside analogs, which act on viral DNA polymerase, HPIs intervene earlier in the replication cycle, enabling effectiveness across multiple stages of infection and bypassing the need for intracellular activation. Notably, HPIs do not rely on viral TK activity for activation, meaning that drug activation does not require prior HSV infection of the host cell. Despite these advantages, there have been reports of HSV developing resistance to HPIs [63]. Taken together, despite advanced clinically available anti-HSV drugs have limitations, including low oral bioavailability, short half-life, inability to prevent recurrent infections, and the potential emergence of drug-resistant strains with long-term use [64]. Vaccine development efforts have yet to yield a clinically effective HSV vaccine. Therefore, novel antiviral agents with distinct mechanisms of action remain a critical need. Marine organisms, with their vast chemical diversity, offer a promising source for the discovery of new anti-HSV compounds. Another major concern in HSV infection is the clinical manifestation of the disease. As a lytic virus, HSV induces cytopathic effects in infected epithelial or neuronal cells, leading to host cell lysis to release viral particles. This process triggers a robust local inflammatory response, characterized by infiltration of neutrophils, macrophages, and T cells, as well as the release of proinflammatory cytokines such as TNF-α, IL-1β, and IL-6. The resulting inflammation contributes directly to the formation of painful vesicular lesions and tissue damage at the site of infection [65]. Therefore, an ideal antiviral strategy would not only inhibit viral entry and replication but also modulate the inflammatory response to reduce lesion severity and associated pain, addressing both the virological and symptomatic aspects of HSV disease.

## 3. Promising Targets for HSV Infections

**Figure 4:**
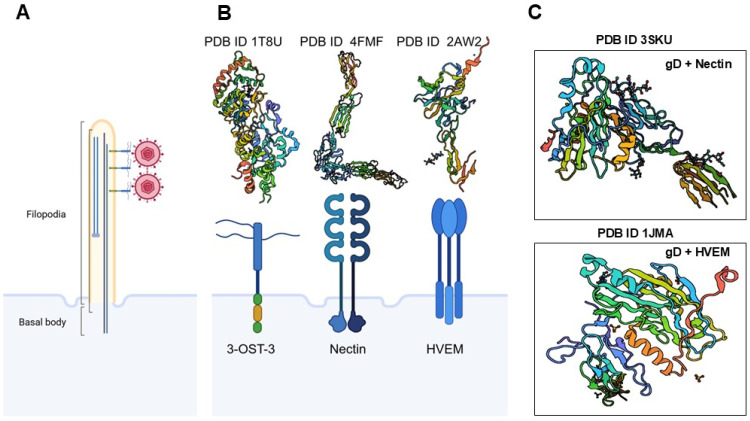
Molecular determinants of HSV-1 attachment and entry. Before infection, HSV-1 virions engage in “surfing” behavior along membrane bound actin-rich filopodia, guided by interactions with heparan sulfate, to reach the cell body (**A**). Viruses exploit pre-existing or dynamically induced filopodia to facilitate their access to host cells, particularly at mucosal epithelial surfaces. These actin-rich protrusions serve as conduits that guide viral particles towards the cell body, enhancing the efficiency of viral attachment and entry. This mechanism leverages the host’s cytoskeletal machinery to promote viral dissemination and infection. The three prototypical receptors for HSV-1 glycoprotein D (gD)—3-O-sulfated heparan sulfate, nectin-1, and herpesvirus entry mediator (HVEM) (**B**) and their crystal structures form PDB data are illustrated. Additional crystal structures of HSV-1 gD interactions with nectin-1 and HVEM are shown from the PDB data set (**C**). The roles of other gD receptors, such as cellular integrins and PILR-α, were not discussed due to space constraints. Created in BioRender. Tiwari, V. https://BioRender.com/1o2gdcj (accessed on 31 December 2025).

Multiple strategies have been developed to target HSV infection, beginning even before virus enters the host cell [66,67,68,69]. Advances in our understanding of molecular interactions between virus and host cell, receptor tropism, viral entry mechanisms, and receptors involvement during cell-to-cell spread have identified several promising targets to prevent infection and viral propagation. HSV virions are enveloped particles containing 17 envelope proteins, including 12 glycoproteins [70]. Among these, gB, gD, gH, and gL are essential for membrane fusion and viral entry [71,72]. Glycoprotein B (gB) plays a central role in mediating virus binding to virus host cell fusion with the plasma membrane [69]. The gB initially binds heparan sulfate proteoglycans (HSPGs) on the cell surface or activated filopodia to facilitate attachment and cooperates with gD and the gH/gL complex to drive membrane fusion and entry (Figure 4A) [73]. Additionally, gB interacts with gH/gL to mediate viral budding and nucleocapsid release [74]. Small molecules, including guanidine-modified pyrimidine derivatives and the oligonucleotide ODN5652, have been reported to inhibit gB-mediated entry, underscoring its potential as a therapeutic target [75]. The pleiotropic glycoprotein D (gD), a homodimer of ~394 amino acids, engages multiple cellular receptors including nectin-1, HVEM, and 3-O-sulfated HS which vary between cell types (Figure 4B,C) [66,76]. Interaction of gD with the gH/gL complex is essential for membrane fusion [77]. Therapeutic strategies targeting gD include monoclonal antibodies (e.g., m27f) that bind the pre-fusion domain to inhibit fusion [78], sulfated gallic acid glucoside (SPGG) to block viral adsorption and entry in the primary cultured cells derived from human eye donors [79], and high-affinity RNA adapters that prevent HSV invasion [80]. The major virus capsid protein VP5 (ICP5) has also been reported as a potential antiviral target. VP5 plays a major role during assembly of capsomeres with other viral capsid proteins such as VP19C, VP23, and VP26 [81], relying on interactions with scaffold proteins and other viral components (VP22a, UL25, VP26, ICP35) for capsid formation [82,83]. The N-terminal hydrophobic residues of VP5 are critical for capsid assembly [84], and VP5 mediates nuclear transport via the dynactin cofactor [85]. siRNAs targeting VP5 or VP23, as well as small molecule inhibitors like Dynasore that disrupt VP5-dynamin interactions, significantly impair HSV replication [85,86]. Similarly, host factors such as heat shock protein 90 (Hsp90), a conserved molecular chaperone involved in protein folding, transport, and stress responses [87], are critical for multiple stages of HSV infection, including viral protein folding, capsid transport, and nuclear localization of viral DNA polymerase [88]. Hsp90 inhibition blocks nuclear egress, viral assembly, and neuronal entry by modulating F-actin reorganization [89,90,91]. Viruses depend on the host cellular machinery for replication and have evolved multiple strategies to manipulate it, creating an intracellular environment conducive to their proliferation and survival. One such strategy involves viral mimicry of key host regulatory proteins to hijack cellular processes. Members of the *Herpesviridae* family encode conserved herpesvirus protein kinases [92], which share functional similarities with host cyclin-dependent kinases (CDKs); hence, they are often referred to as viral CDK-like kinases [93]. CDKs are central regulators of cell cycle progression, transcription, apoptosis, and neuronal function [94]. In HSV infection, CDK1, CDK2, and CDK7 are required for efficient replication in non-neuronal cells, whereas CDK2 plays a critical role in viral reactivation within neurons [95]. Pharmacological inhibition of CDKs disrupts immediate-early, early, and late viral gene expression, thereby reducing HSV replication. For instance, BMS-265246, a selective CDK inhibitor, effectively impairs multiple stages of HSV-1 replication [96]. Moreover, CDK inhibition suppresses replication of multiple other herpesviruses emphasizing CDKs as broad-spectrum antiviral targets [97,98,99].

**Figure 5:**
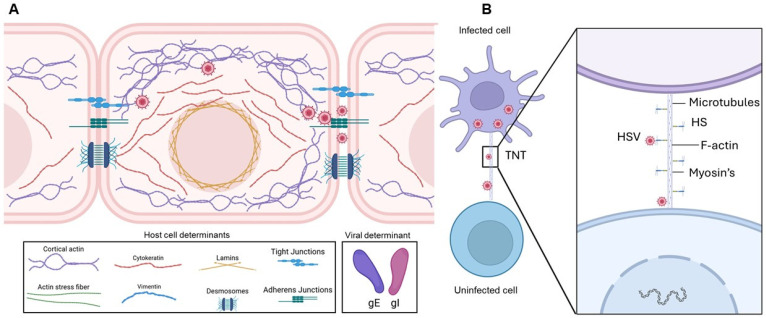
Molecular determinants of HSV-1 cell-to-cell spread. (**A**) HSV-1–infected cells accumulate virions that can infect neighboring cells. During this process, the virus disrupts adherens junctions, liberating nectin-1, which facilitates virus cell-to-cell transmission. Viral glycoproteins gE/gI contribute to the promotion of viral spread. Cell-to-cell junctions, together with heparan sulfate, help maintain barrier integrity and regulate adhesion; however, it remains unclear whether additional junctional openings enhance heparan sulfate’s role in viral spread. Actin and intermediate filaments also support intracellular viral trafficking, contributing to efficient virus dissemination. (**B**) Role of tunneling nanotubes (TNTs) in long-distance HSV spread. HSV-infected cells induce TNT formation to connect with neighboring cells, facilitating viral transfer over distances of 20–100 μm in a short time. Microtubules provide structural rigidity and support cargo transport within TNTs, while molecular motor myosin mediates viral movement through these structures. Heparan sulfate (HS), abundantly expressed on TNTs, likely promotes cell-to-cell viral transfer. Wingless and Int-1 (Wnt) signaling is proposed to play a central role in cytoskeletal remodeling, regulating TNT formation and function. Created in BioRender. Tiwari, V. https://BioRender.com/0agvjp4 (accessed on 31 December 2025).

**Figure 6:**
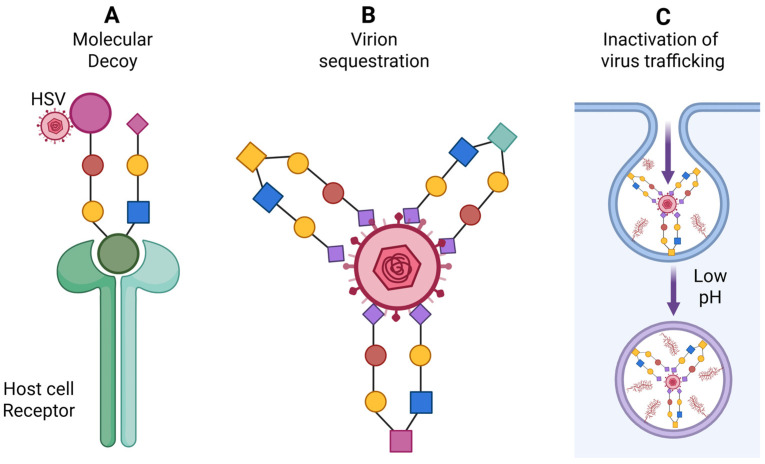
Significance of marine-derived analogs as anti HSV agents. (**A**) Analogs act as molecular decoys interfering with HSV attachment and entry into host cells. (**B**) The analogs exhibiting high affinity for HSV envelope glycoproteins, leading to virion clustering or neutralization and thereby blocking viral entry. (**C**) Analogs mimetics may also disrupt intracellular viral trafficking by trapping virions within the endocytic pathway and inhibiting the low-pH–dependent steps required for viral genome uncoating. Created in BioRender. Tiwari, V. https://BioRender.com/zkcvmhj (accessed on 31 December 2025).

**Figure 7:**
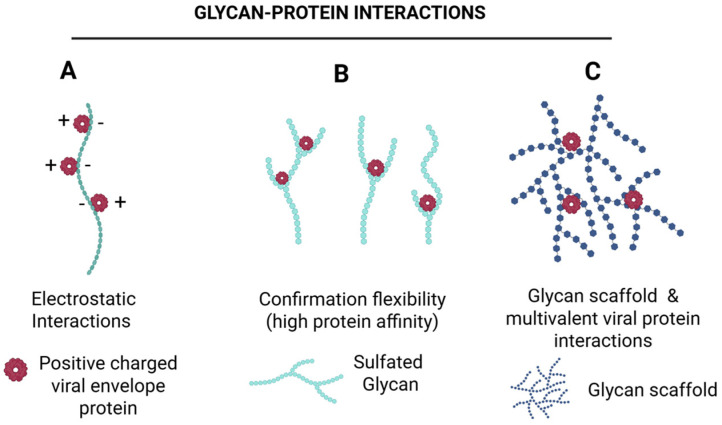
Sulfated glycans enhance protein binding through complementary mechanisms. Sulfated glycans promote protein interactions via—(**A**) electrostatic attraction between negatively charged sulfates and positively charged protein residues, (**B**) conformational effects, where sulfation optimizes glycan geometry for enhanced access to protein binding pockets, and (**C**) glycan scaffold, which enables multivalent interactions, increasing binding avidity and efficiency. Together, these mechanisms illustrate how sulfated glycans function as versatile modulators of protein recognition. Created in BioRender. Tiwari, V. https://BioRender.com/3wkd830 (accessed on 31 December 2025).

HSV employs multiple strategies for spreading, including disruption of adherent-based cadherin junctions to expose nectin-1 for cell-to-cell transmission (Figure 5A) and actin-rich tunneling nanotubes for long-distance travel (Figure 5B). Remarkably, the potential of marine-derived compounds to modulate these alternative pathways remains unexplored. Targeting the early stages of HSV infection continues to represent a highly promising therapeutic strategy. Existing entry inhibitors predominantly function through: (i) heparan sulfate (HS) mimetics or glycomimetics that competitively block viral attachment; (ii) HS-targeted agents that prevent viral adsorption; (iii) compounds that directly bind viral glycoproteins to inhibit membrane fusion; and (iv) sulfated glycans capable of neutralizing the virus (Figure 6). Mechanistically, sulfated glycans confer distinct functional advantages in protein recognition (Figure 7). They enhance protein interactions through three complementary mechanisms such as electrostatic attraction, sulfation-induced conformational effects and the glycan scaffold or extracellular matrix (ECM)–mediated presentation, which supports multivalent interactions and markedly increases protein binding avidity and efficiency. Collectively, these mechanisms underscore the role of sulfated glycans as versatile and dynamic modulators of protein–glycan interactions. Building on this framework, our recent studies demonstrated that marine-derived sulfated glycans, particularly FCS-Ib and Rhamnan sulfate, act via multiple complementary mechanisms to impede HSV entry, including potent virus neutralization upon pre-exposure, effectively disrupting the initial stages of infection [46]. These findings highlight the untapped therapeutic potential of marine glycans in combating HSV infections.

## 4. Marine-Derived Natural Compounds Against HSV Infections

Marine organisms, including sponges, ascidians, seaweeds, and associated microorganisms, are rich sources of antiviral compounds such as polysaccharides, lectin, terpenoids, nucleosides, alkaloids, and peptides (Table 1). Vidarabine (9-β-D-arabinofuranosyladenine, ara-A), a nucleoside derived from spongothymidine and spongouridine isolated from the marine sponge *Tethya crypta*, showed notable antiviral activity. However, its use was later discontinued due to lower efficacy and higher toxicity compared to acyclovir (Zovirax) [100]. Seaweed is a rich source of bioactive natural products with diverse biological activities, including antiviral properties [101]. Marine algae are classified into macroalgae and microalgae. Macroalgae, which grow in coastal regions, include red, brown, and green algae, whereas microalgae inhabit deep-sea water columns, sediments, and coastal habitats, including diatoms, dinoflagellates, brown flagellates, and cyanobacteria. These algae produce a variety of metabolites that have shown promise as anti-HSV agents. Sulfated polysaccharides from red algae are among the most potent anti-HSV agents, primarily acting by inhibiting viral attachment to host cells [51]. Carrageenans, sulfated polysaccharides from red algae, exhibit variable antiviral effects depending on the virus type, including HSV-1 and HSV-2 [102,103]. Krylova et al. demonstrated that carrageenans inhibit HSV infection by binding to viral glycoprotein gD, thereby preventing virus–cell interactions [104]. In vivo studies also showed that carrageenans significantly reduce HSV-2 vaginal infection in murine models [105]. Polyelectrolyte complexes (PECs) composed of carrageenan and chitosan were reported to inhibit early stages of HSV infection more effectively than Carrageenans, highlighting the potential of combination strategies in anti-HSV drug development [106,107]. In addition to polysaccharides, other red algae–derived compounds display potent antiviral effects. Griffithsin (GRFT), a mannose-binding lectin, inhibits HSV-2 secretion and cell-to-cell transmission post-infection [108]. Similarly, the glycolipid sulfoquinovosyl diacylglycerol (SQDG) exhibits strong antiviral activity against both HSV-1 and HSV-2, with IC_50_ values below 50 µg/mL [109]. Overall, red algae derived compounds including sulfated polysaccharides, lectins, and glycolipids demonstrate significant anti-HSV activity, with efficacy often influenced by molecular weight, degree of sulfation, and structural characteristics. Similarly, polysaccharides from green algae, such as ulvans, have been reported to inhibit various viruses, including enterovirus EVA71 and HSV [110], Lopes et al. demonstrated that the sulfated polysaccharide SU1F1 from green algae exhibits potent anti-HSV activity, particularly against HSV-1, with efficacy increasing alongside the degree of sulfation [111]. In addition to polysaccharides, small molecules from green algae also display antiviral activity. The indole alkaloid caulerpin, isolated from marine seaweed *Caulerpa* species, demonstrated strong in vitro anti-HSV effects with an IC_50_ of 1.29 μg/mL, surpassing the activity of ACV, and may inhibit multiple stages of the viral replication cycle [112]. Similarly, ethanolic extracts of freshwater *Spirogyra* spp., containing terpenoids, alkaloids, and essential oils, effectively inhibited HSV-1 infection with an IC_50_ of 2.17 μg/mL [113]. Overall, green algae derived polysaccharides, and small molecules represent promising candidates for novel anti-HSV therapeutics, with antiviral activity influenced by sulfation level, molecular structure, and compound class. The brown algae are also known endogenous producers of a variety of polysaccharides, including alginates, fucoidans, and laminarans, which exhibit diverse therapeutic properties with relatively low toxicity [114,115,116]. Fucoidans from brown seaweed *Nizamuddinia zanardini* have demonstrated potent anti-HSV-2 activity, primarily by inhibiting early stages of the viral life cycle [42]. Similarly, two fucoidans SHAP-1 and SHAP-2 extracted from brown algae *Sargassum henslowianum* showed strong antiviral activity against both HSV-1 and HSV-2, with IC_50_ values below 0.9 μg/mL [115]. Other brown algae polysaccharides have also been reported to interfere with HSV-1 entry, while water extracts from certain brown algae block replication events post-entry [117]. The cyclic GMP–AMP synthase (cGAS)–Stimulator of Interferon Genes (STING) signaling axis is a central component of the innate antiviral immune response [118]. Acting as a cytosolic DNA sensor, cGAS recognizes both exogenous viral DNA and aberrant endogenous DNA, catalyzing the production of cyclic GMP–AMP (cGAMP), which in turn activates STING. This activation triggers downstream signaling cascades involving TANK-binding kinase 1 (TBK1) and Interferon Regulatory Factor 3 (IRF3), ultimately driving the transcription of type I interferons (IFNs) and other antiviral effector genes. Emerging evidence indicates that laminaran, a β-1,3-glucan derived from marine brown algae, can potentiate cGAS–STING signaling, thereby amplifying type I IFN responses and enhancing cellular antiviral defenses (Table 2) [116]. These findings highlight laminaran as a promising immunomodulatory candidate for therapeutic intervention against viral infectious diseases.

In addition to polysaccharides, terpenoids and glycolipids from brown algae exhibit significant anti-HSV activity. The diterpenoid dolabelladienetriol (D1), isolated from marine brown seaweed *Dictyota pfaffii*, inhibits HSV-1 infection in vitro and in vivo in a dose-dependent manner, comparable to acyclovir [119]. The glycolipid sulfoquinovosyl diacylglycerols (SQDG) from Brazilian brown seaweed *Sargassum vulgare* shows strong activity against HSV-1 and HSV-2 in vitro [120]. Other diterpenes, including 10,18-trihydroxy-2,6-dolabelladiene and dihydroxydolasta-1,7-diene, appear to inhibit early events of HSV-1 replication without affecting adsorption or penetration [121]. Similarly, diterpenes hidroxydidichotoma isolated from Brazilian brown algae *Dictyota pfaffii* and *Dictyota menstrualis* also demonstrates significant dose- and MOI-dependent inhibition of HSV-1 replication, highlighting the potential of brown algae terpenoids as candidates for anti-HSV drug development [121]. On the other hand, microalgae are also a rich source of bioactive compounds, including lipids, pigments, peptides, polysaccharides, minerals, and vitamins, many of which exhibit significant antiviral activities, including anti-HSV effects. The sulfated polysaccharide Calcium spirulan (Ca-SP), isolated from a blue-green algae *Spirulina platensis*, has been shown to inhibit replication of several enveloped viruses, including HSV-1 (Table 2) [122]. Notably, Ca-SP also inhibited entry of Kaposi sarcoma-associated herpesvirus/human herpes virus 8. In the clinical model of herpes exacerbation, the prophylactic effect of a Ca-SP and microalgae extract containing cream was superior to that of acyclovir cream [122]. Additionally, lectins from blue-green algae have demonstrated potent antiviral effects. A newly identified lectin significantly inhibited plaque formation in HSV-1–infected Vero cells, likely by acting directly on virions and blocking the initial stages of infection [123]. Our previous study also demonstrated that Cyanovirin-N, a microalgae-derived lectin, effectively prevents HSV-1 infection by blocking membrane fusion mediated by HSV envelope glycoproteins [124].

**Table 1:** Algae-derived marine compounds with anti-HSV activity.

Marine Source	Compound/ Class	HSV Type	Stage Targeted	Mechanism of Action	IC_50_/EC_50_ (Approx.)	References
Red algae	Carrageenans (κ, λ, ι)	HSV-1 HSV-2	Attachment Entry	Bind viral gB/gC/gD and host heparan sulfate, preventing absorption	0.4–5.6 µg/mL	[102,103,104,105]
Brown algae	Fucoidans (SHAP-1, SHAP-2)	HSV-1 HSV-2	Early infection	Inhibit viral attachment and penetration in a sulfation-dependent manner	<0.9 µg/mL	[115]
Green algae	Ulvans/sulfated polysaccharides	HSV-1	Entry/ Replication	Suppress HSV replication block entry	0.036–8.5 µg/mL	[46,110,111]
Red algae	Griffithsin (lectin)	HSV-2	Post-entry spread	Blocks cell-to-cell transmission by binding viral envelope glycans	~230 nM EC_50_ (~0.035 µg/mL)	[108]

**Table 2:** Marine microbial metabolites exhibiting anti-HSV activity.

Marine Source	Compound/ Class	HSV Type	Stage Targeted	Mechanism of Action	IC_50_/EC_50_ (Approx.)	References
Mollusk	Abalone hemocyanin	HSV-1	Entry	Binds viral gB/gC/gD, preventing virion attachment/penetration	1.8–5 µg/mL	[125]
Brown algae	Laminaran	HSV-1	Host response	Activates cGAS–STING, type I interferon signaling, indirectly suppressing HSV replication	20–60 µg/mL	[116]
Cyanobacteria (*Spirulina* sp.)	Calcium spirulan	HSV-1	Entry/ Replication	Blocks viral penetration/early replication, reported superior topical efficacy to ACV †	0.4–1.3 µg/mL	[122]

† ACV; Acyclovir.

## 5. Marine Microbes as Emerging Source of Anti-Herpesvirus Therapeutics

Marine microorganisms have long been recognized as prolific producers of structurally diverse and pharmacologically active natural products. Over the last decade, this ecological reservoir has yielded a growing number of antiviral candidates [126], including several secondary metabolites from marine bacteria and fungi with potent activity against herpesviruses (Table 3) [127]. Although the exploration of marine-derived antivirals targeting HSV remains comparatively limited, current findings reveal a landscape rich in chemical novelty and unique mechanisms of action.

Marine fungi, in particular, stand out as exceptional sources of small molecules, peptides, and complex metabolites with antiviral potential. Among these, peniterphenyl A—isolated from the deep-sea fungus *Penicillium* SCSIO 41030 blocks HSV adsorption and entry by directly engaging the viral gD protein, offering a mechanistic alternative to classical nucleoside analogs such as acyclovir [128]. Other fungal metabolites, including emodin A and neoechinococcin D from sponge-associated fungi, demonstrate near-complete inhibition of HSV-1 [128]. Cyclic peptides such as simplicilliumtide J and aspergillipeptide D further extend this antiviral repertoire, effectively suppressing multiple HSV-1 strains, including acyclovir-resistant isolates [129,130]. Notably, aspergillipeptide D (APD) exerts its activity by downregulating glycoprotein gB and disrupting its trafficking between the Golgi apparatus and endoplasmic reticulum, thereby impairing virion assembly and egress [131]. Additionally, Halovir A, a virion-inactivating hexapeptide from the marine-derived fungus *Scytalidium*, exhibited potent anti-HSV activity [132].

**Table 3:** Marine microbial metabolites exhibiting anti-HSV activity.

Marine Source	Compound/ Class	HSV Type	Stage Targeted	Mechanism of Action	IC_50_/EC_50_ (Approx.)	References
Marine fungi (*Penicillium* sp.)	Peniterphenyl A	HSV-1	Entry	Direct interaction with viral gD, inhibiting membrane fusion	1.6–3.2 µM	[128]
Marine Fungi (*Aspergillus* sp.)	Aspergillipeptide D	HSV-1 ACV-R †	Late replication	Impairs gB intracellular trafficking and virion assembly	9–14 µM	[131]
Marine bacteria (*Bacillus* sp.)	γ-Poly(glutamic acid)	HSV-1 HSV-2	Replication/ Inflammation	Inhibits viral replication, suppresses TNF-α and IL-1β expression	15–45 µg/mL	[133]

† ACV-R; Acyclovir Resistant.

Deep-sea fungi and bacteria continue to expand this chemical diversity. A butenolide derivative from *Streptomyces koyangensis* SCSIO 5802 exhibits anti-HSV-1 activity (EC_50_ = 25.4 μM), while compounds from *Simplicillium obclavatum* EIODSF 020, including simplicilliumtide J and verlamelins A and B, inhibit HSV-1 with IC_50_ values of 14.0–16.7 μM [130]. Anthraquinones such as aspergilols H and I and coccoquinone A from *Aspergillus versicolor* SCSIO 41502 show even greater potency (EC_50_ = 3.12–6.25 μM) [134]. Similarly, tetramic acid derivatives from the deep-sea fungus *Trichobotrys effuse* DFFSCS021—trichobotrysins A, B, and D exhibit antiviral activity comparable to or surpassing acyclovir (IC_50_ = 3.08–9.37 μM) [135]. Hydroxamate-containing cyclopeptides from *Acremonium persicinum* SCSIO 115, including acremonpeptides A and B and Al(III)–acremonpeptide D, show moderate HSV inhibition (EC_50_ = 8.7–16 μM) [136]. Fungi associated with gorgonian corals also contribute novel scaffolds, such as aspergillipeptides D and E from *Aspergillus* sp. SCSIO 41501 (IC_50_ = 9.5–19.8 μM), with aspergillipeptide D retaining activity against acyclovir-resistant strains [137]. Additional metabolites from *Aspergillus terreus* SCSGAF0162 such as 12α-dehydroxyisoterreulactone A, arisugacin A, isobutyrolactone II, and aspernolide A likewise show substantial antiviral effects (IC_50_ = 6.34–28.9 μg/mL) [138]. From the Baltic Sea, the macrolide balticolid (IC_50_ = 0.45 μM) and the highly potent naphthalenone derivatives balticols A–F, particularly balticol E (IC_50_ = 0.01 μg/mL), further highlight the breadth of fungal antiviral chemistry [139]. Marine bacteria also contribute significantly to anti-HSV discovery, often through metabolites that target distinct stages of HSV infection. γ-Poly(glutamic acid) (γ-PGA) from *Bacillus horneckiae* inhibits early replication and modulates inflammatory cytokines such as TNF-α and IL-1β, whereas peptide A-3302-B disrupts later stages of the viral cycle [133]. Digolide O, an amphotericin derivative from marine *Streptomyces*, displays potent anti-HSV-1 activity with minimal cytotoxicity [42]. Additionally, several extracellular polysaccharides EPS1-B3-15, EPS1-T14, and EPS2 significantly inhibit HSV-2 replication in peripheral blood mononuclear cells [140]. Collectively, these studies underscore the immense and largely untapped potential of marine microbes as reservoirs of structurally diverse antiviral compounds. Many of these metabolites operate through mechanisms wholly distinct from conventional nucleoside analogs, offering new therapeutic avenues and expanding the chemical landscape for future anti-HSV drug development.

### Bioactive Metabolites from Marine Invertebrate with Anti-HSV Activity

Marine invertebrates, including sponges, tunicates, echinoderms, and mollusks, are rich sources of bioactive compounds with antiviral potential. Unlike marine vertebrates, these organisms rely solely on innate immune systems and produce secondary metabolites to defend against pathogens. Sponges (*Phylum Porifera*) are simple, sessile animals with porous bodies that filter water for nutrients and produce diverse metabolites such as chloroalkane diterpenes from *Raspailia bouryesnaultae*, which inhibit HSV-1 replication by over 50% in the KOS strain and more than 70% in the 29R strain [141]. Tunicates (*Subphylum Tunicata*), or sea squirts, are sac-like filter-feeding animals covered by a cellulose-like tunic; they produce cyclic peptides known as didemnins (A–C) that inhibit various RNA and DNA viruses in vitro with IC_50_ values below 0.1 μM, and ethanol extracts from tunicates also inhibit HSV-2 by targeting the viral DNA polymerase UL30 gene [142]. Echinoderms (*Phylum Echinodermata*), including sea urchins, sea stars, and sea cucumbers, are marine animals with radial symmetry, a water vascular system, and a calcareous endoskeleton; they produce echinochrome analogues (EAMA and EAMB) that reduce HSV-1 plaque formation by binding viral gD protein, and triterpenoid glycosides and ethanol extracts from sea cucumbers interfere with HSV attachment with minimal cytotoxicity [143]. Mollusks (*Phylum Mollusca*), such as snails, clams, octopuses, and abalones, are soft-bodied animals often protected by calcareous shells; they rely on innate effectors like antimicrobial peptides and hemocyanin, the latter of which from *Haliotis rubra* inhibits HSV-1 in vitro by binding viral glycoproteins gB, gD, and gC, preventing viral entry without affecting later stages of replication [51]. Collectively, these invertebrates produce structurally diverse secondary metabolites with antiviral, anti-inflammatory, and antitumor properties, representing valuable leads for the development of novel anti-HSV therapeutics.

## 6. Molecular Targets and Antiviral Mechanism of Marine Derived Compounds Anti-HSV Agents

Marine-derived compounds, including algal polysaccharides and secondary metabolites from diverse marine organisms, exhibit antiviral activity against HSV by targeting multiple stages of the viral life cycle and enhancing host antiviral immune responses. The HSV life cycle involves sequential processes including viral adsorption, entry, capsid uncoating, replication, and viral assembly and release [46]. Several marine compounds exert direct virucidal effects by binding viral particles and disrupting their structural integrity. For example, carrageenan, a negatively charged sulfated polysaccharide, interacts with HSV glycoproteins gB and gC, inducing conformational changes that inactivate virions, likely via formation of a virion–carrageenan complex that blocks envelope sites necessary for adsorption [102]. As mentioned above, the marine peptide Halovirs A from *Scytalidium* fungi directly binds HSV particles, resulting in inactivation of HSV-1 virions. Beyond direct inactivation, marine compounds interfere with viral adsorption, a process primarily mediated by interactions between viral glycoproteins and host cell surface glycosaminoglycans (GAGs) such as heparan sulfate. High-molecular-weight carrageenan can block viral binding to host cells, whereas λ-carrageenan from *Gigartina skottsbergii* binds HSV receptors to prevent attachment. Sea urchin spinochromes act as competitive inhibitors of the pleotropic envelope glycoprotein D (gD), and octadecanoic acid ether ester from sea cucumber disrupts virus–receptor interactions, thereby reducing successful viral attachment [42].

Marine compounds also inhibit viral entry by preventing membrane fusion, endocytosis, or capsid uncoating. Sulfated polysaccharides from brown algae, such as alginic acid, effectively block HSV entry, while Peniterphenyl A derived from deep sea derived *Penicillium* sp. binds gD to inhibit membrane fusion [117]. Similarly, a marine-derived compound—Abalone hemocyanin from *haliotis rubra* (Sea snail) interacts with glycoproteins gB, gC, and gD, preventing entry without affecting late-stage replication (Table 2) [144]. Following entry, several marine-derived compounds interfere with viral replication by targeting viral enzymes or intracellular replication processes. Water extracts from brown algae inhibit post-entry replication, and carrageenan can suppress intermediate replication events. Caulerpin, a secondary metabolite from green algae *Caulerpa* sp., inhibits both α- and β-stage replication [112], whereas ethanol extracts of *Styela plicata* target HSV-2 DNA polymerase UL30 [142]. Interestingly Aspergillipeptide D—a cyclic pentapeptide isolated from the marine gorgonian-derived fungus *Aspergillus* sp. did not affect HSV-1 early infection events, including viral inactivation, attachment and penetration but it does impact the late expression of glycoprotein gB and causes gB mislocalization in the cellular compartments in the endoplasmic reticulum and Golgi apparatus impairing viral replication via unique mechanism [131]. Besides blocking HSV infection, marine compounds have also shown potential in interfering with HSV replication. In addition to these direct antiviral actions, marine compounds can enhance host antiviral immunity, thereby promoting viral clearance. Fucoidan from brown algae enhances in vitro and in vivo anti-HSV responses, and carrageenan improves natural killer cell activity and lymphocyte proliferation, strengthening innate and adaptive immune defenses [145]. γ-Poly(glutamic acid) from marine *Bacillus* not only blocks early viral replication but also upregulates proinflammatory cytokines such as TNF-α and IL-1β, further contributing to viral elimination [133], marine compounds can modulate signaling pathways including NF-κB, which regulates expression of antiviral cytokines, providing an indirect yet potent mechanism to inhibit HSV infection. Collectively, these observations highlight the multifaceted potential of marine-derived compounds to disrupt HSV infection at multiple stages, combining direct virucidal action, inhibition of adsorption and entry, suppression of replication, and augmentation of host immune responses, thereby offering promising avenues for antiviral drug development.

## 7. Modulating HSV-Associated Inflammation Using Marine-Derived Bioactive Compounds

Glycosylation is among the most pervasive post-translational modifications of proteins, shaping a wide spectrum of biological functions from proper protein folding and structural stability to viral attachment and escape from antibody neutralization [146]. The glycans decorating host-cell receptors further fine-tune viral binding, thereby influencing the efficiency of virus entry. As glycosylation research technologies continue to advance, glycan-based strategies are emerging at the forefront of antiviral drug and vaccine development, capturing growing scientific attention [147]. HSV infections, which can affect the skin, mucosa, cornea, or central nervous system, are characterized by recurrent lesions and robust local inflammatory responses. Both viral replication and immune-mediated tissue damage contribute to lesion formation, pain, edema and delayed healing. Current antiviral therapies primarily target viral replication but do not sufficiently attenuate virus-induced inflammation, a major determinant of disease severity and patient morbidity. Therefore, there is a critical need for therapeutic agents that combine antiviral efficacy with modulation of host inflammatory pathways. Marine organisms, particularly echinoderms such as sea cucumbers, sea urchins, and starfish, provide a rich source of structurally diverse bioactive molecules with potent anti-inflammatory properties, offering promising scaffolds for the development of adjunctive therapies to mitigate HSV-associated immunopathology.

### 7.1. Inflammatory Pathways in HSV Pathogenesis

**Figure 8:**
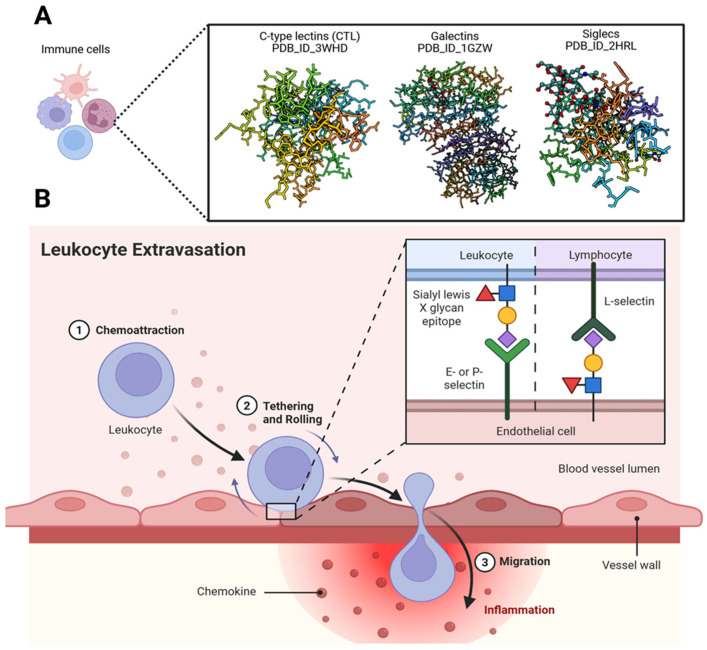
Significance of glycans in immune regulation. (**A**) Mammalian glycans are tightly regulated within the immune system, and their interactions with glycan-binding proteins (GBPs) modulate diverse cellular mechanisms alongside other molecular signals. These interactions play essential roles in both innate and adaptive immunity. GBPs decode subtle structural differences between host and pathogen glycans, enabling precise discrimination during immune surveillance. Immune cells expressing GBPs—such as C-type lectins (CTLs), galectins, and Siglecs—are shown using structures retrieved from the Protein Data Bank. By recognizing and binding specific carbohydrate determinants, GBPs function as pattern recognition receptors (PRRs) for a wide range of pathogens. (**B**) The leukocyte migration cascade across the endothelium is regulated by glycan-binding proteins such as selectins, along with glycosylated adhesion molecules including ICAM-1. These interactions play critical roles in leukocyte recruitment and transmigration, and they represent promising therapeutic targets for controlling chronic inflammation. Created in BioRender. Tiwari, V. https://BioRender.com/m77b6nt (accessed on 31 December 2025).

HSV infection activates innate immune sensors, including Toll-like receptors (TLRs) and cytosolic pattern-recognition receptors (PRRs), initiating intracellular signaling cascades such as mitogen-activated protein kinases (MAPKs), phosphoinositide 3-kinase (PI3K), Janus kinase-signal transducer and activator of transcription (JAK-STAT), and NF-κB [148]. These signaling pathways culminate in transcriptional upregulation of pro-inflammatory mediators, including tumor necrosis factor-alpha (TNF-α), interleukins IL-1β and IL-6, inducible nitric oxide synthase (iNOS), and cyclooxygenase-2 (COX-2) [20]. The resulting cytokine and chemokine milieu promotes leukocyte infiltration, apoptosis, and tissue edema, processes in which glycans play a significant regulatory role (Figure 8). By targeting these conserved signaling nodes, echinoderm-derived bioactive compounds have the potential to attenuate HSV-induced inflammation and limit lesion exacerbation.

#### Anti-Inflammatory Mechanisms of Echinoderm-Derived Compounds and Mechanistic Relevance to HSV Infection

Sea cucumbers are rich in polysaccharides, triterpenoid glycosides, peptides, and lipid fractions, all of which have been reported to modulate inflammatory signaling. Sulfated polysaccharides, such as fucosylated chondroitin sulfate and fucoidan, inhibit NF-κB and JNK activation, suppress pro-inflammatory cytokine expression, and reduce iNOS and COX-2 activity in immune-stimulated models [20]. Triterpenoid glycosides, including holothurin A and echinoside A, attenuate macrophage infiltration and suppress prostaglandin E2 (PGE2) biosynthesis via inhibition of ERK/cPLA2/COX-1 pathways [20]. Peptide hydrolysates derived from enzymatic digestion of sea cucumber proteins have demonstrated inhibition of TLR4/MyD88-dependent NF-κB activation, resulting in decreased TNF-α, IL-1β, and IL-6 expression and enhanced anti-inflammatory cytokine secretion (IL-10, TGF-β) [20]. Lipid fractions enriched in eicosapentaenoic acid (EPA) promote macrophage polarization toward the M2 phenotype and reduce pro-inflammatory signaling in adipose and hepatic tissue. Collectively, these mechanisms target critical nodes implicated in HSV-mediated immunopathology, suggesting a potential role in mitigating lesion-associated tissue damage and inflammatory pain.

Sea urchins contribute additional bioactive molecules capable of modulating inflammation relevant to HSV infection. Pigments such as Echinochrome A attenuate immune cell infiltration, downregulate pro-inflammatory cytokines, and promote M2 macrophage polarization in murine colitis models, indicative of potential efficacy in mucocutaneous HSV lesions [144]. Sulfated polysaccharides and peptides from sea urchins inhibit prostanoid and leukotriene synthesis and suppress NF-κB signaling, closely aligning with the molecular pathways activated during HSV reactivation [114]. Starfish-derived extracts, although less extensively characterized, demonstrate cytokine-modulatory activity and inhibition of inflammatory signal transduction, reinforcing the pharmacological potential of echinoderms for targeting virus-induced inflammation. The anti-inflammatory effects of echinoderm-derived compounds converge on key signaling hubs activated during HSV infection, including NF-κB, MAPK, and TLR4/MyD88 pathways [149]. These agents also reduce the expression of pro-inflammatory enzymes (iNOS, COX-2) and cytokines (TNF-α, IL-1β, IL-6), while promoting macrophage polarization toward the anti-inflammatory M2 phenotype [149]. By modulating these pathways, echinoderm bioactive could attenuate lesion severity, reduce leukocyte-mediated tissue injury, and accelerate mucocutaneous healing, complementing the antiviral activity of standard therapeutics without suppressing immune-mediated viral clearance.

### 7.2. Translational Implications with Marine Anti-Inflammatory Profiles

**Figure 9:**
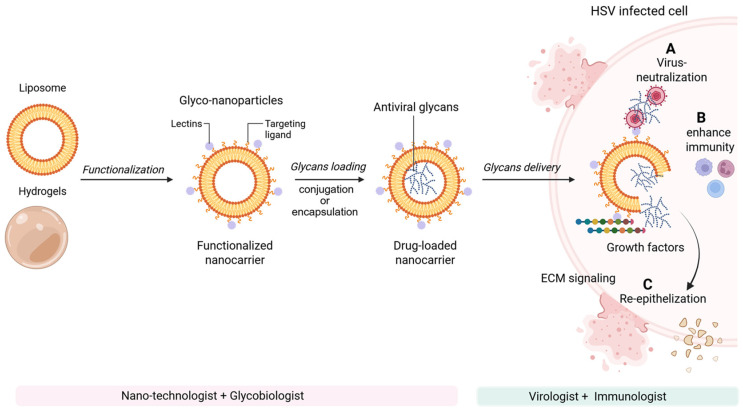
Glycan-based nanoengineering strategies for next-generation antiviral therapeutics. Nanoengineered glycan platforms exert antiviral and regenerative effects through multiple complementary mechanisms: (A) direct viral neutralization by blocking viral attachment, fusion, or entry; (B) modulation and enhancement of host immune responses, including improved antigen presentation and innate immune activation; and (C) targeted interactions with immune and stromal cells to establish localized growth factor gradients that promote extracellular matrix remodeling and tissue regeneration, thereby restoring tissue function following HSV-mediated lytic cell damage. Although glyco-nanocarrier systems remain in early stages of antiviral development, their demonstrated success in oncology highlights strong translational potential as targeted anti-inflammatory, antiviral, and wound-healing therapies for virus-infected cells and tissues. Advancing glycan-based antiviral therapeutics will require a highly collaborative, multidisciplinary approach integrating expertise in glycobiology, nanotechnology, immunology, virology, and translational medicine. Created in BioRender. Tiwari, V. https://BioRender.com/5a03dt4 (accessed on 31 December 2025).

Glycan-based nanoengineering offers a multifunctional approach for next-generation antiviral therapeutics by integrating direct viral neutralization with host-directed immune and regenerative effects (Figure 9). Despite compelling preclinical evidence, translation into HSV therapies remains constrained by challenges in pharmacokinetics, bioavailability, toxicity, and sustainable sourcing. Nevertheless, mechanistic insights provide a strong framework for therapeutic development. Echinoderm-derived bioactives, including polysaccharides, glycosides, peptides, and lipids from sea cucumbers, sea urchins, and starfish, represent an underexplored reservoir of dual-acting antiviral and anti-inflammatory agents. These compounds modulate key inflammatory pathways, including NF-κB, MAPK, and TLR4/MyD88 signaling, suppress pro-inflammatory cytokine production, and promote anti-inflammatory macrophage polarization, processes directly relevant to HSV-associated immunopathology [149]. Future translational studies using standardized HSV infection models—particularly brain, skin, corneal, and vaginal organoid based systems, will be essential to optimize dosing, evaluate safety, and advance marine-derived glyco-nanocarriers as adjunctive therapeutics targeting both viral replication and lesion-associated inflammation.

## 8. Structural and Computational Strategies for Marine Derived Antivirals

The integration of high-resolution structural biology with advanced computational modeling is redefining mechanism-based antiviral discovery from marine natural products, particularly for HSV. Techniques such as single-particle cryo-EM, synchrotron crystallography, multidimensional NMR, and Hydrogen/Deuterium Exchange Mass Spectrometry (HDX-MS) are now resolving the conformational landscapes of key HSV entry determinants—including gD, gB, gH/gL, and host receptors such as nectin-1, HVEM, and 3-O sulfated heparan sulfate (Figure 4) [68]. These approaches delineate metastable prefusion post-fusion transitions, transient receptor-engagement intermediates, and concealed allosteric pockets that may be preferentially targeted by structurally elaborate marine metabolites. Time-resolved cryo-EM and rapid-exchange mass spectrometry further capture microsecond-to-millisecond fluctuations within gD–receptor interfaces and gB fusion loops [149], illuminating how marine derived compounds scaffolds could stabilize non-productive conformers or allosterically suppress the fusogenic cascade required for membrane entry.

Concurrently, AI-driven modeling including AlphaFold-multistate structural ensembles, graph neural networks, molecular dynamics informed docking, deep generative chemistry, and machine learning-based viral fitness prediction enables rapid identification and optimization of marine derived antiviral leads [150,151]. These tools estimate ligand engagement across dynamic conformers, model perturbations to fusion energetics, and guide scaffold evolution to enhance potency, selectivity, and physicochemical properties. Machine-learning pipelines for biosynthetic gene cluster mining and de novo metabolite prediction further expand access to sulfated, polyoxygenated, and macrocyclic marine scaffolds with innate capacity to disrupt HSV attachment or fusion.

Together, these structural and computational methodologies form an iterative discovery loop in which experimentally derived viral conformational ensembles constrain AI-based predictions, while in silico analyses refine hypotheses on allosteric hotspots, receptor-blocking strategies, and conformational trapping mechanisms. This integrated framework shifts marine derived compounds-based HSV antiviral development from empirical screening to a precise, mechanistically informed platform capable of yielding next-generation inhibitors that target the dynamic molecular choreography governing herpesvirus entry and dissemination.

## 9. Future Challenges with Marine-Derived Antivirals

Marine-derived compounds, including sulfated polysaccharides, peptides, and alkaloids show substantial antiviral potential, yet several barriers must be overcome before they can progress to clinically viable therapeutics. A major challenge lies in their pharmacokinetics and bioavailability. For instance, sulfated polysaccharides often exhibit poor oral absorption due to their high molecular weight, structural heterogeneity, and susceptibility to enzymatic degradation in the gastrointestinal tract. Their pharmacodynamic properties are further influenced by immunomodulatory activity and dependence on the route of administration, complicating dosage optimization and systemic delivery [152]. Although the use of fluorescent and near-infrared labeling has improved the study of biodistribution and metabolism, standardized, highly sensitive in vivo tracking methods remain limited [152].

Another critical limitation is the scarcity of clinical evidence. Most investigations rely on in vitro assays or animal models, and little is known about their efficacy, safety, and pharmacokinetics in humans [45]. In addition, structure–activity relationships governing antiviral potency remain only partially understood, hindering rational optimization of these molecules [153]. While oral delivery remains problematic, topical applications such as vaginal gels have shown promise for preventing localized infections like HSV-2, indicating that alternative administration routes may be more feasible [154].

Beyond pharmacological considerations, practical challenges also impede translation. Sustainable sourcing of marine organisms, efficient extraction and purification workflows, and adherence to stringent safety standards are required for scalable production [155]. Moreover, environmental variability can alter compound composition, affect reproducibility, and complicate regulatory evaluation. Addressing these pharmacological, mechanistic, and logistical challenges will be essential to advance marine-derived antivirals from experimental systems toward clinical deployment.

## 10. Conclusions

Marine-derived polysaccharides, particularly algal sulfated glycans, represent a vast and underexploited reservoir of antiviral agents with demonstrated activity against HSV. These compounds exert antiviral effects through multiple complementary mechanisms, including inhibition of viral attachment, entry, and replication, as well as modulation of host innate immune responses. Their capacity to reduce inflammation and enhance antiviral immunity suggests relevance not only for primary infection but also for limiting viral latency and reactivation, which are central challenges in HSV pathogenesis. Among these bioactives, rhamnan sulfate has emerged as a particularly powerful anti-HSV candidate. Beyond direct inhibition of HSV-1, rhamnan sulfate displays broad host-directed activities, including suppression of inflammatory signaling pathways, modulation of NF-κB and growth factor interactions, enhancement of endothelial barrier integrity, and attenuation of vascular and metabolic dysfunction. This convergence of antiviral, immunomodulatory, vascular, and metabolic effects is rarely observed within a single molecular scaffold and highlights its unique therapeutic breadth. While these compounds show exceptional promise, remaining challenges related to structural heterogeneity, standardization, and bioavailability present clear opportunities for innovation and refinement. Addressing these barriers through rigorous structural characterization, optimized formulation strategies, and scalable production approaches will be critical for clinical advancement. Future investigations should also evaluate efficacy against clinical and acyclovir-resistant HSV isolates using physiologically relevant human models. Collectively, marine polysaccharides and rhamnan sulfate in particular offer strong translational potential and a compelling foundation for the development of next-generation therapeutics against HSV and other persistent viral infections.

## Data Availability

The original contributions presented in this study are included in the article/supplementary material. Further inquiries can be directed to the corresponding author(s).
